# Synteny and phylogenetic analysis of paralogous thyrostimulin beta subunits (GpB5) in vertebrates

**DOI:** 10.1371/journal.pone.0222808

**Published:** 2019-09-19

**Authors:** Krist Hausken, Berta Levavi-Sivan

**Affiliations:** Department of Animal Sciences, Robert H. Smith Faculty of Agriculture, Food, and Environment, The Hebrew University of Jerusalem, Rehovot, Israel; Universite de Rouen, FRANCE

## Abstract

At some point early in the vertebrate lineage, two whole genome duplication events (1R, 2R) took place that allowed for the diversification and sub-/neo-functionalization of the glycoprotein hormones (GpHs). All jawed vertebrates possess the GpHs luteinizing hormone (LH), follicle stimulating hormone (FSH), and thyroid stimulating hormone (TSH), each of which are heterodimers with a common alpha subunit and unique beta subunits. In 2002, a novel glycoprotein hormone named thyrostimulin was described to have unique GpA2 and GpB5 subunits that were homologous to the vertebrate alpha and beta subunits. The presence of GpA2 and GpB5 in representative protostomes and deuterostomes indicates their ancestry in the GpH family. There are several reports of GpH subunit evolution, but none have included GpA2 and GpB5 for species in each major vertebrate class. Thus, we addressed the ancestry of two paralogous GpB5 subunits (GpB5a and GpB5b) that were previously only recognized in two teleost species. Our search for orthologous GpB5a and GpB5b sequences in representative vertebrates and phylogenetic analysis, in addition to the currently published evolutionary scenarios of the GpH family, supports that GpB5a and GpB5b are paralogs that arose from the first vertebrate whole genome duplication event (1R). Syntenic analysis supports lineage specific losses of GpB5a in chondrichthyes, basal actinopterygians, and tetrapods, and retention in coelacanth and teleosts. Additionally, we were unable to identify GpA2 transcripts from tilapia mRNA, suggesting that this species does not produce heterodimeric thyrostimulin. While the conserved or even species-specific functional role of thyrostimulin or its individual subunits are still unknown in vertebrates, the analyses presented here provide context for future studies on the functional divergence of the GpH family.

## Introduction

In vertebrates, the glycoprotein hormone family includes luteinizing hormone (LH), follicle stimulating hormone (FSH), thyroid stimulating hormone (TSH), and chorionic gonadotropin (CG; in placental mammals only). These glycoprotein hormones are non-covalently linked heterodimers comprised of a common alpha subunit (GpHα/GpA1/CGA) and distinct, yet paralogous, hormone-specific beta subunits [1]. In any one species of jawed vertebrates, the primary amino acid sequence of the alpha subunits are identical because they are encoded by the same gene; however all beta subunits are transcribed from separate genes and therefore confer biological specificity [2]. Glycoprotein hormone subunits belong to the superfamily of cystine-knot forming proteins. The alpha and beta subunits have 10 and 12 conserved cysteine residues, respectively, which participate in forming a core intra-subunit cysteine knot and subunit heterodimerization [1, 3, 4]. The mature hormones are secreted from the pituitary gland and act at specific G-protein coupled receptors in the thyroid (TSH receptor (R)) or gonads (LHR/FSHR) to regulate metabolism, development, gametogenesis, steroidogenesis, and ovulation.

A fifth member of the GpH family was identified in 2002 from human pituitary and named thyrostimulin for its ability to activate the human TSHR and increase serum thyroxine concentrations [5, 6]. The subunits of thyrostimulin, GpA2 and GpB5, are homologous and chemically distinct from the traditional GpH alpha and beta subunits. It was previously thought that the GpH family arose within the vertebrate lineage. However, the discovery of GpA2 and GpB5 introduced major insight into the evolution of the GpH family because they have been identified in representative protostomes and deuterostomes, including: the nematode, *Caenorhabditis elegans* [7]; insects such as the fly, *Drosophila melanogaster* [7], and the silkworm, *Bombyx mori*; the mollusk, *Aplysia californica* [8]; protochordates, *Ciona* and *Branchiostoma* [9]; and representatives of every vertebrate class [7, 9–12]. The diversification and sub-functionalization of the GpHs arose from two rounds of whole genome duplication events (1R, 2R) that took place early in the vertebrate lineage [13].

Thyrostimulin has only been examined in mammals [5–7, 14, 15], a frog [7], elephant shark [10], sea lamprey [12], and invertebrates (reviewed in [16]). Despite the specific functions of GpHs in modern vertebrates, there is limited data supporting a conserved or ancestral role of thyrostimulin in vertebrates. Given the conservation of the physiological roles of modern GpHs across vertebrates, the functional role of thyrostimulin may be obsolete or lineage specific. For example, GpB5 knockout mice exhibited limited phenotypic differences from wildtype mice, suggesting that thyrostimulin is either not required for viability or that genetic compensation (possibly from the other GpHs) exists [17]. However, in sea lampreys, which lack LH, FSH, and TSH, thyrostimulin was hypothesized to be a major player in the hypothalamic-pituitary-gonad/thyroid axis [12]. To date, there have been no reports of teleost thyrostimulin, a group which boasts the highest diversity of vertebrate species.

Phylogenetic analyses of the glycoprotein hormone family in vertebrates have been comprehensive except for teleost GpB5 sequences. Previously, zebrafish was found to contain two paralogous GpB5 subunits, named GpB5a and GpB5b, and along with pufferfish were the only organisms where GpB5a was identified [18]. These paralogous GpB5 subunits were proposed to have arisen as a result of 1R and lost in the tetrapod lineage [19]. Given that GpB5a was not described in any non-teleost species at the time, we decided to search for orthologs and perform phylogenetic and synteny analyses in order to revisit the ancestry of GpB5 homologs in 30 species representative of each major vertebrate class, providing a current view of the vertebrate GpH family.

## Materials and methods

### Animals

Nile tilapia (*Oreochromis niloticus*) were kept and bred in the fish facility unit at the Hebrew University (Rehovot, Israel) in 500-L tanks at 26°C with 14L:10D photoperiod. Fish were fed daily with commercial fish pellets (Raanan Fish Feed, Miluot, Israel). This study, the method of euthanization, and all experimental procedures were approved by the Hebrew University administrative panel for laboratory animal care.

### Cloning

Three adult female tilapia were anesthetized in 0.01% phenoxyethanol and quickly decapitated, and then their brain, pituitary, eye, muscle, ovary, heart, kidney, intestine, liver, thyroid, spleen, gill, and stomach were dissected and frozen in liquid nitrogen. Ten juvenile tilapia were anesthetized and frozen whole in liquid nitrogen. Tissues or whole juvenile fish were homogenized in Trizol reagent (Invitrogen, Carlsbad, CA) with a stainless steel bead at 25Hz for 2 minutes in a Tissuelyser II system (Qiagen, Hilden, Germany) and total RNA was extracted by adding chloroform, then washed, dried, and dissolved in ultra-pure water. First strand cDNA was synthesized from 1 μg of total RNA using the Verso cDNA synthesis Kit (Thermo Scientific, Waltham, MA) with 3:1 (v/v) random hexamers:poly-dT primers. The cDNA mastermix was added after the RNA was initially denatured at 70°C/5min, then the reaction was incubated at 42°C/1hr, 95°C/2min, and finally 10°C/5min. GpB5a and GpB5b were amplified from 25ng pituitary cDNA with 200nM of GpB5a_F (ATGCACCTCCACCGTATCG) and GpB5a_R (TCAGAAGGTCTCACACTCTGTG), and GpB5b_F (ATGACCCTTCAAAGGAGACAGC) and GpB5b_R (TCACACAGAGGTTATGCATTCAGTAG), respectively using the Advantage II polymerase system (Promega, Madison, WI). GpB5a reactions were initially denatured at 95°C/1min followed by 35 cycles of 95°C/30s denaturation, 57°C/30s annealing, and 68°C/1min extension; GpB5b was amplified under the same conditions except the annealing temperature was 63°C. A single adenine was added to each PCR product by incubating the final reaction at 70°C/10min. Bands of expected sizes were extracted from a 1% agarose gel and purified on a Promega Wizard column (Promega, Madison, WI). The gel products were ligated into a vector by the TOPO-TA cloning kit (Thermo Scientific), transformed into competent DH5α cells, and incubated on LB-ampicillin plates overnight at 37°C. Single colonies were selected and grown in liquid cultures overnight, and the following day plasmid DNA was extracted using miniprep columns (Qiagen, Hilden, Germany). All products were sequenced with T7 and Sp6 primers by the Life Science Core Facility at the Weizmann Institute of Science (Rehovot, Israel).

### Multiple sequence alignment and phylogenetic analysis

The amino acid sequences for GpA2, GpB5a, and GpB5b from 30 species representative of each vertebrate class/order were identified by orthologous pBLAST/BLAT of tilapia or human sequences on NCBI, UCSC, or Ensembl databases.

Tetraodon, zebrafish, tilapia, and coelacanth sequences for GpA2, GpB5a, and GpB5b were ran as tBLASTn queries [20] against the Atlantic sturgeon (*Acipenser oxyrhincus*; [21], Yebra-Pimentel *et al*. *in preparation*) draft genome. Query hits with a bitscore lower than 70 were eliminated and the resulting contigs were joined with the genome using Galaxy (usegalaxy.org) in order to extract the sequences, which were verified by NCBI BLAST. The signal peptides for each of the amino acid sequences were predicted using SignalP3.0 [22] and removed for multiple sequence alignment (MUSCLE) using MEGA7. Amino acid sequences were aligned and the percent identity was calculated using MatGAT [23].

Evolutionary analyses were conducted in MEGA7 [24]. The evolutionary history was inferred by using the Maximum Likelihood method based on the JTT matrix-based model [25]. The tree with the highest log likelihood (-3269.51) is shown. Initial tree(s) for the heuristic search were obtained automatically by applying Neighbor-Join and BioNJ algorithms to a matrix of pairwise distances estimated using a JTT model, and then selecting the topology with superior log likelihood value. A discrete Gamma distribution was used to model evolutionary rate differences among sites (5 categories (+G, parameter = 1.6398)). The rate variation model allowed for some sites to be evolutionarily invariable ([+I], 5.68% sites). The analysis involved 56 amino acid sequences. All positions with less than 95% site coverage were eliminated. That is, fewer than 5% alignment gaps, missing data, and ambiguous bases were allowed at any position. There were a total of 88 positions in the final dataset.

### Conserved synteny analysis

We examined genes upstream and downstream of GpA2 and GpB5b for representative species of each vertebrate class. Gene organization was initially obtained by using BLAST in Genomicus version 91 with tilapia GpB5a or GpB5b [26]. Missing orthologs from the Genomicus results were identified using NCBI, Ensembl, and UCSC genome browsers. When whole genome data was unavailable or incomplete, BLAST was used in an attempt to annotate the species-specific scaffold/contig where GpA2 or GpB5b were found. For European eel, we identified syntenic genes to GpB5a and GpB5b by BLAST against the more recently released genome [27]. Species that lacked these flanking genes were excluded from the synteny analysis. Chromosome/scaffold/contig location and reference numbers can be found in S1 and S2 Tables.

## Results

### GpA2 and GpB5 sequences

Searches for orthologous GpB5a subunits sequences resulted in the identification of GpB5a in selected teleosts and the basal sarcopterygian, coelacanth. Zebrafish GpB5a (NCBI NC_007118) is likely a pseudogene because it does not have a start codon within the first exon and the C-terminal end of exon 2 is missing conserved cysteine residues.

**Fig 1:**
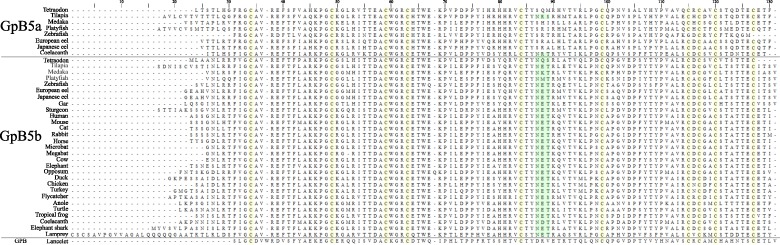
Multiple sequence alignment of GpB5 sequences. GpB5a and GpB5b amino acid sequences (signal peptides removed) from 30 species that represent each vertebrate class were aligned using MUSCLE. GpB5a sequences are shown on top, GpB5b in the middle, and lancelet GPB on the bottom. Conserved cysteine residues are highlighted with yellow boxes, and the conserved N-glycosylation motif is highlighted in green boxes.

The cloned full-length cDNAs of tilapia GpB5a and GpB5b encode 129 and 139 amino acid proteins with putative 18 and 29 amino acid signal peptides, respectively. Amino acid alignment of tilapia GpB5a and GpB5b with orthologous GpB5a and GpB5b sequences shows conservation of ten cysteine residues and one N-glycosylation site for both subunits (Fig 1). We failed to clone GpA2 from different tissues of adult tilapia or pooled whole juvenile tilapia with varying primer sets and PCR conditions. BLASTn was performed with the genomic-derived cDNA sequence for GpA2 on RNA sequencing datasets for tilapia pituitary (SRR5331652) using NCBI’s sequence read archive (SRA) database and no hits were found, providing further evidence that GpA2 is not expressed in tilapia pituitary.

Sturgeon GpA2 was identified on relatively short contigs (507971, 421967, and 189431). The contig sequences overlapped, and so were compiled and organized based on alignment with orthologous GpA2 sequences, resulting in a coding sequence (92 amino acids) for the final two out of three exons; the first exon was not found by any BLAST queries, or by attempting to combine hits using AUGUSTUS gene product prediction. There were no hits for GpB5a in sturgeon. Based on amino acid alignment, we assume the GpB5 hit against the sturgeon genome is GpB5b. Both exons of sturgeon GpB5b were identified on contig 198525 (6643-7316bp, reverse).

European eel GpA2, GpB5a, and GpB5b sequences were initially identified with the newer genome [27], but these sequences were incomplete or incorrect relative to the previously released assembly [28]. Therefore, we used the more complete sequences from the older genome for multiple-sequence alignment and phylogenetic analysis, but used the newer genome for synteny analysis because of the larger scaffold sizes.

### Multiple sequence alignment, phylogenetic, and syntenic analyses of GpA2 and GpB5

No stark differences were found between GpB5a and GpB5b sequences that could clearly indicate their identities from one another in any species, except that only coelacanth and tilapia GpB5a had a conserved N-glycosylation motif (N-X-S/T (Fig 1). Generally, GpB5a lacks some acidic amino acids that are conserved in GpB5b sequences that may or may not be involved in electrostatic interactions with a receptor. The exception is coelacanth GpB5a, which aligns more with tetrapod GpB5b sequences than the teleost GpB5a sequences.

**Fig 2:**
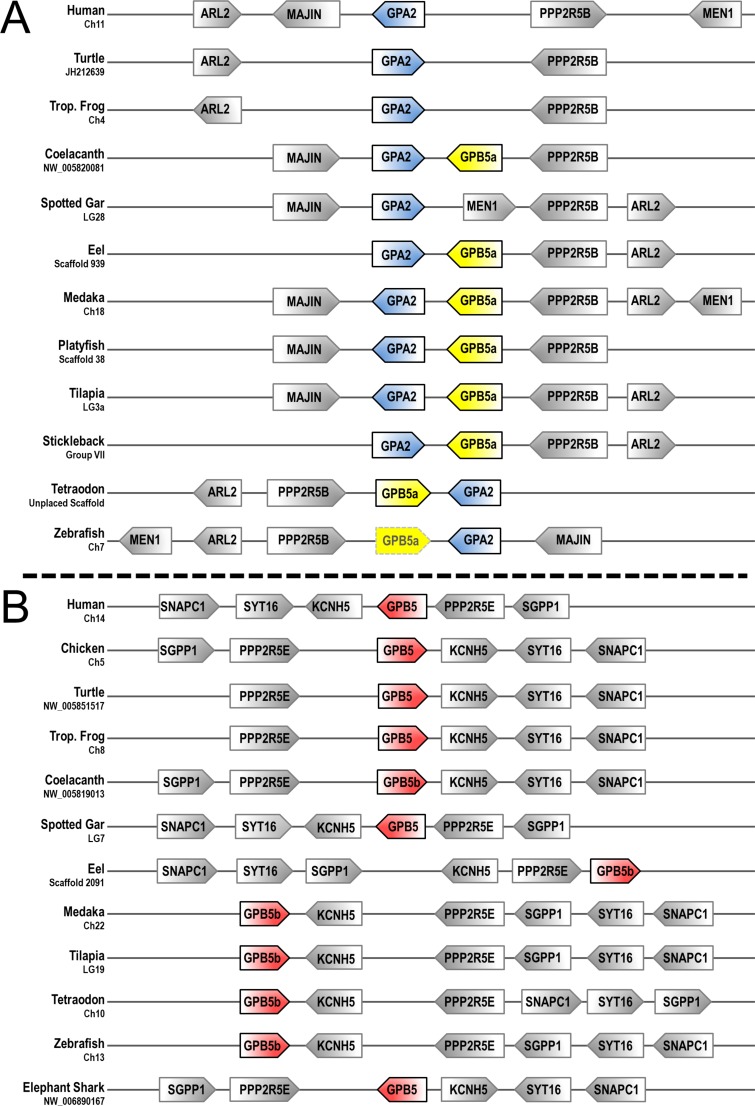
**Patterns of conserved synteny for A) GpA2 and B) GpB5b, and their flanking syntenic genes.** Chromosomes are oriented 5’-3’ and chromosome/scaffold identifiers are listed under the organism name. GpA2 is highlighted in blue, GpB5a in yellow, and GpB5b in red. Zebrafish GpB5a is shown with a broken box because it appears to be a pseudogene.

Given the high sequence similarity between GpB5a and GpB5b, we performed a syntenic analysis in order to address the identities of these paralogues in different species (Fig 2). When comparing the gene neighborhood relative to GpB5a in different species it was always immediately adjacent to GpA2, whereas GpB5b was found on a different chromosome/scaffold.

**Fig 3:**
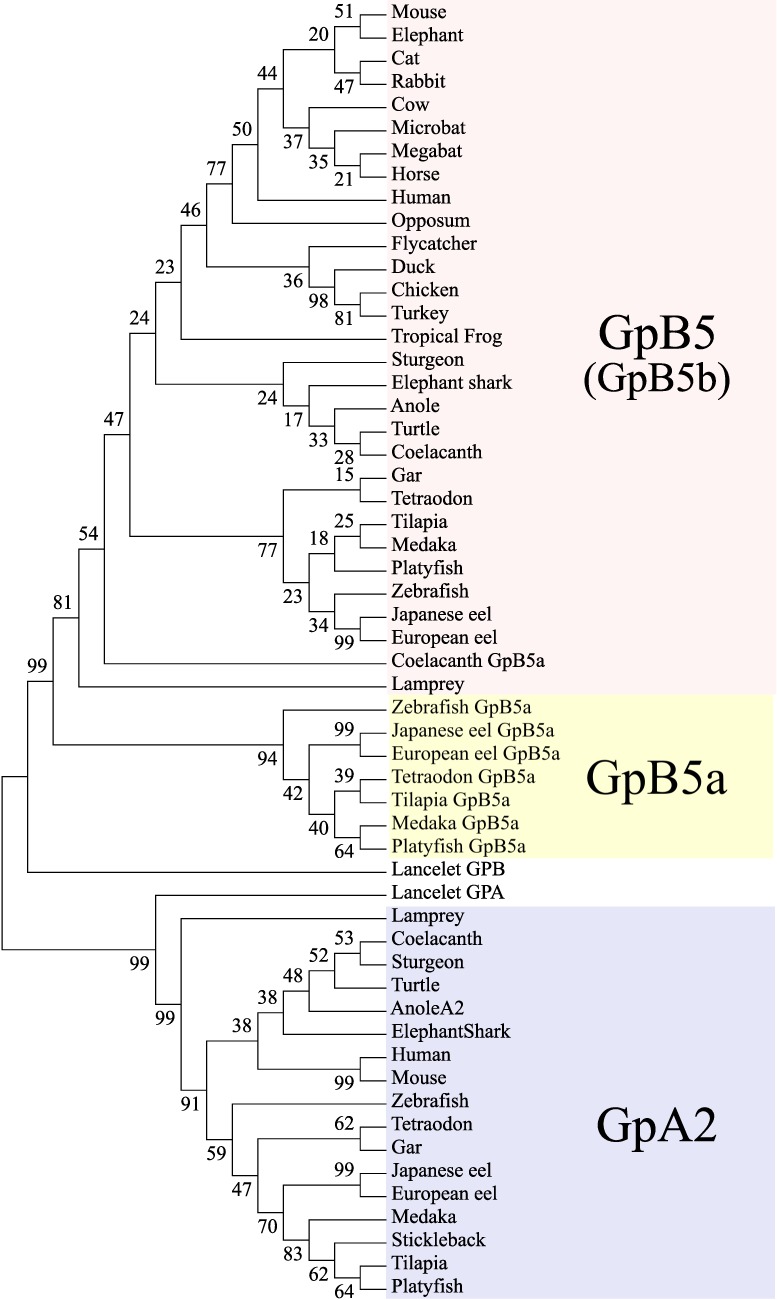
Maximum likelihood phylogenetic tree depicting the evolutionary relationships of GpA2 and GpB5. GpA2 sequences are highlighted with a blue background, GpB5 sequences with red, and the teleost group of GpB5a sequences are in yellow. Coelacanth GpB5a is labelled to distinguish it from GpB5b, although both fall into the GpB5b clade. Lancelet GPB and GPA are included as outgroups in this unrooted tree.

Our phylogenetic analysis shows that teleost GpB5a sequences form a monophyletic sister clade with all other GpB5 sequences, suggesting that these sequences diverged prior to 3R (Fig 3). Coelacanth GpB5a was placed within the GpB5b clade, probably because it has a higher amino acid sequence similarity with other vertebrate GpB5b sequences compared to orthologous (teleost) GpB5a sequences (S3 Table).

## Discussion

Our results confirm that teleosts possess two paralogous GpB5 subunits, GpB5a and GpB5b, which were likely products of 1R due to the presence and synteny of GpB5a in coelacanth and high support of the branching clades. Coelacanth GpB5a is a syntenic match to the teleosts, despite the phylogenetic analysis supporting its placement in the GpB5b clade. The identification of gar and sturgeon GpA2 and GpB5 subunits from genomic datasets suggested that GpB5a was lost in non-teleost actinopterygian lineages. We are the first to have cloned GpB5a from Nile tilapia mRNA, however GpB5a has yet to be studied outside of a phylogenetic context. Multiple sequence alignment shows that GpB5b has one conserved glycosylation site which is necessary for secretion [29], but since only tilapia and coelacanth possess GpB5a we suggest that in all other species GpB5a is either not translated or secreted. The inability to clone or identify tilapia GpA2 transcripts in publicly available transcriptome databases implies that thyrostimulin is not a functioning pituitary glycoprotein hormone in this fish.

The first reports of the primary amino acid sequences of LH, FSH, and TSH began in the early 1970s in mammals [30–33]. Cloning of these genes by 1990 allowed for subsequent phylogenetic analyses in an attempt to understand their evolution [34–37]. Early phylogenetic analysis proposed that TSHβ and FSHβ formed a sister group to LHβ and CGβ. These two sister groups were paralogous to GTH I and GTH II in fish, which would be reclassified as FSH and LH [38]. In this scenario, the ancestral gene would have duplicated and given rise to LHβ and an ancestral FSHβ/TSHβ, followed by a subsequent duplication separating the FSHβ and TSHβ loci [39, 40]. However, in this analysis the conserved core cysteine residues were not aligned. A new analysis with aligned cysteine residues supported a monophyletic relationship between TSHβ, LHβ, and FSHβ and suggested a direct ancestor for LHβ and FSHβ [41]. The discovery of GpA2 and GpB5 in 2002 redefined the evolution of the GpH family by demonstrating the presence of homologous subunit sequences from invertebrates and human, supporting that an ancestral vertebrate GpHβ duplicated into pre-TSHβ and pre-GTHβ [7, 42]. Most recently, Dos Santos et al. provided strong evidence for this proposed mechanism based on compared synteny and chromosomal mapping of GpH subunit genomic environments in human, chicken, lizard, and amphioxus [19].

**Fig 4:**
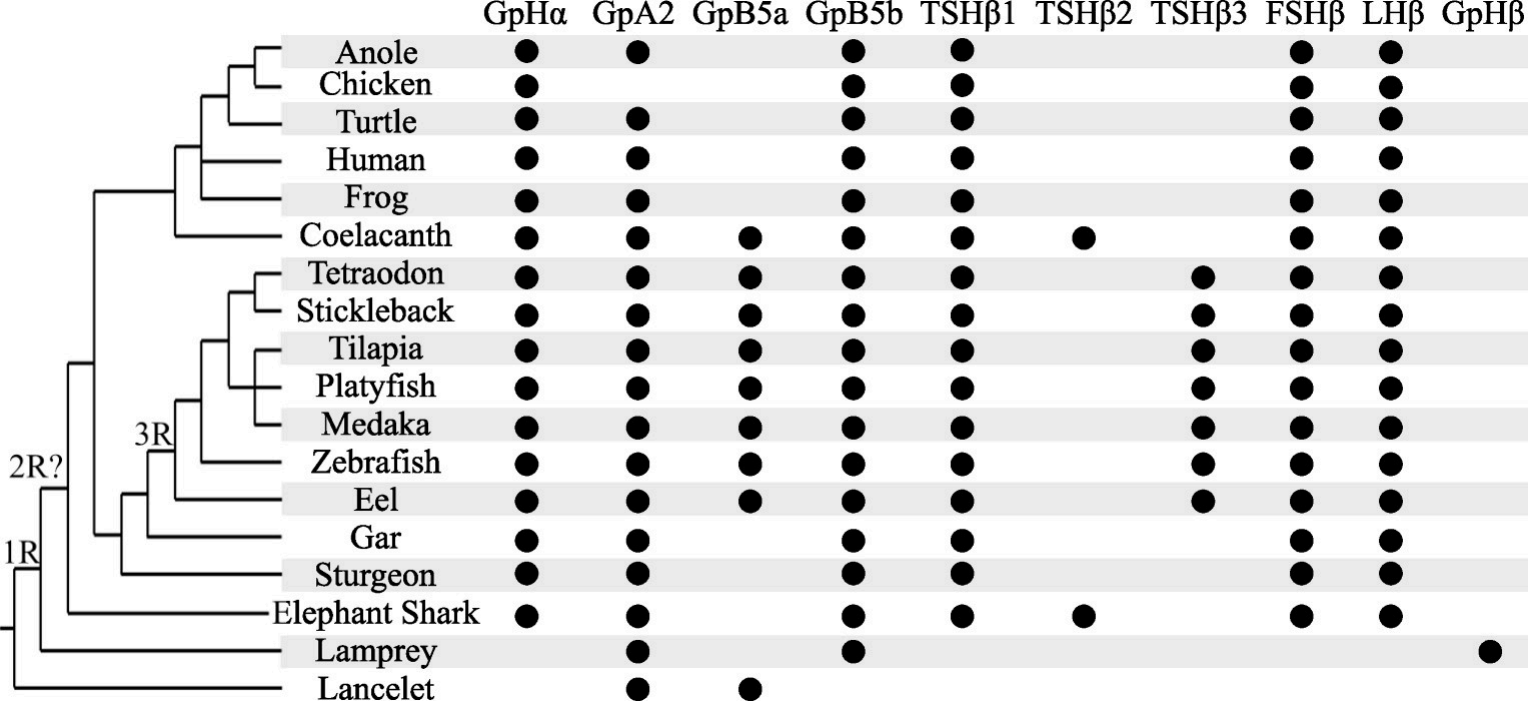
Glycoprotein hormone subunits identified in each vertebrate class to date. On the left is a representative tree depicting the evolutionary relationship of chordates with timing of whole genome duplication events (1R, 2R?, 3R). Homologous glycoprotein hormone genes that have been identified to date (excluding chorionic gonadotropin) are listed across the top, and a black circle indicates the presence of that gene in the specified organism.

Our phylogenetic and syntenic analyses support the existing evolutionary scheme of the GpH protein family by Dos Santos et al. Shared synteny with genes neighboring *gpa2* and *gpb5* suggested that these subunits duplicated prior to 1R from a GPA and GPB locus in an ancestral chordate, resulting in a tetra-paralogon comprised of GpB5-GpA2-GpHα and preGpHβ in the vertebrate ancestor [19]. Therefore, 1R gave rise to GpB5b, GpB5a-GpA2-GpHα, pre-TSHβ, and GtHβ, and the second round of whole genome duplication (2R) would amass the eight GpH subunit paralogs that have all been identified in various vertebrate species: GpB5a-GpA2, GpB5b, TSHβ_1_, TSHβ_2_, LHβ, FSHβ, and GpHα (Fig 4) [10, 19, 43]. It is accepted that all jawed vertebrates descend from a common ancestor that was subjected to 2R, but the precise timing of 2R relative to agnathans, the oldest class of vertebrates, is still debated [44–46]. Lampreys and hagfish (superclass Cyclostomata) are the only extant agnathans. After extensive molecular and biochemical analyses, LHβ, FSHβ, or TSHβ subunits have not been identified in lampreys or hagfish [47]. However, both jawless fish possess “GpHβ” subunits that are ancestral to the LHβ, FSHβ, and TSHβ clades, and while hagfish have the typical GpHα subunit it appears to have been lost in the lamprey lineage [11, 47, 48]. Lampreys possess a single alpha subunit, GpA2, and there is evidence for two lamprey GpB5 transcripts, but the sequence of the second GpB5 is undetermined [12, 19]. From the standpoint of GpH subunit evolution, the presence of these genes in lamprey resembles the repertoire of GpH genes for 1R, suggesting that these agnathan GpHβ subunits are the “GtHβ” in Dos Santos et al.’s model. However, this is unlikely given that there is no evidence of an expected pre-TSHβ gene or pseudogene for any vertebrate to date [11, 47, 49]. Additionally, cyclostomes diverged some 550 million years ago from the vertebrate lineage and several taxa went extinct between the cyclostomes and chondrichthyes, presenting large gaps of time between and within these lineages that create genomic challenges [50]. The limited and ambiguous genomic information available from agnathans complicates our understanding of early vertebrate GpH evolution, and further genomic and functional evidence will be required to elucidate or confirm these mechanisms.

To fully understand the evolution of GpHs, we must also consider the phylogeny of their receptors [51]. GpH receptors (GpHRs) are type A leucine-rich repeat containing G-protein coupled receptors (LGR-A) belonging to the larger seven-transmembrane rhodopsin-like receptor family, and have characteristically large extracellular domains [52]. LGR-A is present in sponges and jellies with origins predating bilaterians, however GpHs are a bilaterian novelty [53]. Invertebrates possess one LGR-A, which was found to be the specific binding partner for thyrostimulin [42, 54]. Lampreys have two LGR-A (lGpH-R I and II) that are activated by the two lamprey glycoprotein hormones (lGpH and l-thyrostimulin), whereas all gnathostomes have three LGR-A that are complimentary to the GpHs (LHR, FSHR, and TSHR) [12, 53, 55]. Single FSHR and LHR genes were identified in a number of gnathostomes, including the elephant shark, whereas LHR duplicated in the actinopterygian lineage prior to 3R (teleost-specific genome duplication) and TSHR duplicated during 3R with teleosts [10, 43, 56, 57]. A specific cognate receptor for thyrostimulin remains unidentified in vertebrates.

Reciprocal structural constraints imposed by the coevolution of paralogous GpHs and their receptors after 1R/2R contributed to the functional specificity of their interactions [58, 59]. Given that thyrostimulin likely co-evolved and interacted with an ancestral GpHR, what is its role in modern vertebrates? The mechanism of action and physiological consequences of thyrostimulin remain largely unknown, and because a *de facto* thyrostimulin has yet to be extracted from blood or tissue, recombinant proteins have been used to characterize all binding, reporter, and *in vivo*/ *in vitro* studies [5, 6, 10, 42, 54]. A wide range of potential physiological functions have been implicated for thyrostimulin, including cancer, immunity, and reproduction (reviewed in [60]). While thyrostimulin is hypothesized to be one of two pituitary GpHs in sea lamprey that function in an endocrine manner [11, 12, 47], there is no apparent conserved endocrine role in the elephant shark or mammals [6, 10]. In the principle thyrostimulin report, the authors proposed a paracrine mechanism where thyrostimulin fine-tunes TSH secretion via TSHR expression in folliculo-stellate cells of the pituitary gland [6]. Thyroidal and ectopic TSHR expression in addition to the wide tissue distribution patterns for GpA2 and GpB5 support the concept of some paracrine mechanism [5, 6, 14]. Previous analysis of initial subunit heterodimerization and physiological dissociation suggests that GpA2 and GpB5 could only form a heterodimer at super-physiological concentrations in circulation, also indicative of a short-term paracrine role instead of an endocrine role [3].

In the initial report, GpA2 and GpB5 subunits were co-expressed as a recombinant heterodimer and named thyrostimulin for its ability to bind and activate the human TSHR, but not LHR or FSHR, and elevate serum thyroxine in mice [6]. It was later found that human TSHR has two high affinity sites for thyrostimulin binding and one binding site for TSH [5]. Thyrostimulin competitively displaced TSH, but the opposite was untrue; this effect was not attributed to differences in affinity, but to the fact that the binding sites for thyrostimulin overlap with and effectively block the site for TSH [5]. However, ligand binding and receptor activation are discrete, non-mutually exclusive events in TSHR signaling. TSHR can be activated by multiple different factors, including constitutively activating mutations, TSHR autoantibody binding, proteolytic cleavage of the extracellular domain, and obviously hormone binding (reviewed in [61]). Hormone binding is perceived by the isomerization of an intra-molecular agonist (a highly conserved peptide sequence located in the C-terminal cysteine box region just preceding the first transmembrane helix) that induces structural changes in the transmembrane helices, resulting in intracellular signaling [62, 63]. The TSHR has also been shown to form higher order structures (homodimers) that demonstrate negative cooperativity, where binding of one receptor decreases the binding affinity of the second receptor [64]. This phenomenon was speculated to permit sensitivity to both low and high concentrations of TSH observed in the blood and pituitary gland, respectively. At the intracellular level, TSHR homodimers activate differential signaling cascades dependent on the number of TSH molecules bound [65]. The physiological consequences of thyrostimulin/TSHR interaction are therefore potentially complex due to the ectopic expression, diverse capacity for activation, and higher order oligomeric properties of the TSHR ([64, 65], and reviewed in [61]).

A key structural and functional feature of GpHs is the ability to form heterodimers. There are conflicting views on the ability for GpA2 and GpB5 to heterodimerize due to the lack of the two C-terminal cysteine residues (the “seatbelt”) of GpB5 relative to the classic GpHs, rendering the accepted mechanism of association impossible [3, 66, 67]. The seatbelt has been demonstrated to be indispensable to stabilize GpH heterodimers at physiological concentrations in the blood (reviewed in [68]). Despite this argument against stable heterodimerization, co-expressed recombinant GpA2 and GpB5 subunits formed heterodimers with [6, 29, 42, 69] or without [5, 12, 14] additional chemical cross-linking agents, suggesting an alternative mechanism of association. Such an alternate mechanism could lower the dissociation rate of the heterodimer, potentially stabilizing it under physiological conditions [12]. Alternatively, GpA2 and/or GpB5 could function in a non-canonical fashion as monomers. GpA2 or GpB5 could not individually displace radiolabeled thyrostimulin from its human TSHR binding sites, or activate rat or elephant shark TSHR [5, 10, 69]. However, GpB5 alone activated the TSHR, albeit at a concentration 100-fold higher than the heterodimer, indicating that binding of the GpB5 monomer can occur [5]. Additionally, branchiostoma GpA2 and GpB5 monomers bound to, but did not activate, LGR1 [5, 54]. Perhaps these monomeric subunits play an essential role as local antagonists of TSHR by modulating its activation potential or outcome, a role that was suggested for the heterodimeric thyrostimulin in its first report [6]. On the other hand, an unidentified interaction with some other receptor could be equally plausible given the meager information available for GpA2 and GpB5 monomers.

Questions remain regarding the structural characteristics of GpB5. Whether GpB5 can function as a monomer or does indeed form a heterodimer with GpA2 would impact its ability to bind and/or activate TSHR in an endocrine or non-endocrine manner. GpB5a was lost in most vertebrate lineages, suggesting that it plays a minor functional role compared with GpB5b. Since GpB5 is conserved in all bilaterians (except hymenopterans) [9], it is assumed to have retained or adapted some essential, yet still unknown role in modern vertebrates. Studies on the role(s) of thyrostimulin and/or GpB5 in actinopterygians are warranted in order to address the functional evolution of thyrostimulin in vertebrates.

## Supporting information

S1 File Vertebrate GpA2 and GpB5 amino acid sequences used in this study in FASTA format. (TXT)

S1 Table Accession numbers of GpA2/GpB5a and syntenic genes in vertebrate species. (XLSX)

S2 Table Accession numbers of GpB5b and its syntenic genes in vertebrate species. (XLSX)

S3 Table Percent identity matrix of GpB5a and GpB5b proteins. (XLSX)
